# Study of Estrogen Receptor and Progesterone Receptor Expression in Breast Ductal Carcinoma In Situ by Immunohistochemical Staining in ER/PgR-Negative Invasive Breast Cancer

**DOI:** 10.5402/2011/673790

**Published:** 2011-07-19

**Authors:** Andrei Dobrescu, Monique Chang, Vatsala Kirtani, George K. Turi, Randa Hennawy, Alexander A. Hindenburg

**Affiliations:** ^1^Division of Oncology/Hematology, Department of Medicine, Winthrop-University Hospital, 200 Old Country Road, Suite 450, Mineola, NY 11501, USA; ^2^Division of Immunohistopathology, Department of Pathology, Winthrop-University Hospital, Mineola, NY 11501, USA; ^3^Department of Pathology, Winthrop-University Hospital, Mineola, NY11501, USA

## Abstract

*Background*. To our knowledge, the hormone receptor status of noncontiguous ductal carcinoma in situ (DCIS) occurring concurrently in ER/PgR-negative invasive cancer has not been studied. The current study was undertaken to investigate the ER/PgR receptor status of DCIS of the breast in patients with ER/PgR-negative invasive breast cancer. *Methods*. We reviewed the immunohistochemical (IHC) staining for ER and PgR of 187 consecutive cases of ER/PgR-negative invasive breast cancers, collected from 1995 to 2002. To meet the criteria for the study, we evaluated ER/PgR expression of DCIS cancer outside of the invasive breast cancer. *Results*. A total of 37 cases of DCIS meeting the above criteria were identified. Of these, 16 cases (43.2%) showed positive staining for ER, PgR, or both. *Conclusions*. In our study of ER/PgR-negative invasive breast cancer we found that in 8% of cases noncontiguous ER/PR-positive DCIS was present. In light of this finding, it may be important for pathologists to evaluate the ER/PgR status of DCIS occurring in the presence of ER/PgR-negative invasive cancer, as this subgroup could be considered for chemoprevention.

## 1. Introduction

Breast cancer evolves from normal epithelium of the terminal duct or lobular unit through a series of increasingly abnormal proliferative lesions beginning with atypical hyperplasia, to premalignant in situ disease, to malignant and increasingly invasive neoplasia [1–3]. In situ carcinoma is characteristically contained within the epithelium, with the basement membrane intact, and without any signs of invasion [4]. Ductal carcinoma in situ (DCIS) is probably a continuum of successive steps of the same process, with increasing malignant potential as the disease progresses from papillary to comedo forms. DCIS originates by proliferation of the ductal luminal cells, which form protrusions into the lumen, called papillary DCIS [5]. These may become more coalescent, leaving a few empty, rounded spaces, known as cribriform DCIS. When the lumen is filled with proliferating cells, it becomes completely obliterated, termed as solid DCIS. Central areas of these ducts undergo necrosis because of the ischemic microenvironment, which results in comedo DCIS [4]. 

ER expression is generally low in normal breast epithelium, except for a small peak in the first week of the menstrual cycle [6]. On average, 80% of atypical ductal hyperplasias are associated with ER overexpression [7–9]. Approximately 75% of low-grade DCIS lesions express ER compared to only 30% of the high-grade DCIS lesions [8–12]. ER positivity is found in up to 60 to 70% of invasive breast cancers [13, 14].

Expression of ER in DCIS alone compared to contiguous DCIS associated with invasive carcinoma has been investigated in the past. One study showed that intraductal carcinoma associated with invasive cancer was more frequently ER-positive compared to DCIS without associated invasion [15].****There was a strong concordance of ER/PgR expression in contiguous DCIS associated with invasive cancer (98%) with virtually all cases being ER/PgR positive.

To our knowledge there is no literature regarding the hormone receptor status of noncontiguous DCIS occurring concurrently in ER/PgR-negative invasive cancer. The current study was undertaken to investigate the hormonal receptor status of DCIS of the breast in patients with ER/PgR-negative invasive breast cancer. 

## 2. Materials and Methods

We reviewed ER/PgR immunohistochemical (IHC) staining of invasive breast cancer cases performed by the pathology department at Winthrop University Hospital from 1995 to 2003. The stain results were as follows: ER+/PgR+ = 358 cases (50.4%), ER+/PgR− = 109 cases (15.3%), ER−/PgR+ = 56 cases (7.9%), and ER−/PgR− = 187 cases (26.3%). Of these, 187 cases of ER-negative/PgR-negative breast cancer were the subgroup of interest for this study. For the purpose of our study, the DCIS component for ER/PgR analyses was required to be at least 1 mm remote from the border of invasive cancer in order to ensure noncontiguity. Cases with DCIS only within the tumor and cases with no residual DCIS were excluded from the study. Given this strict inclusion criteria, 150  women were excluded from the study. The remaining 37 cases, representing 19.7% of the ER/PgR-negative carcinomas and 5.2% of the total group of breast carcinomas, were the subject of ER/PgR status of DCIS that occurs in association with ER/PR-negative invasive breast carcinoma. These were analyzed for ER/PgR status in the DCIS component. 

ER/PgR staining by immunohistochemistry using avidin biotin complex methodology (ABC) with heat-induced epitope retrieval was performed on all cases of invasive breast carcinoma as a routine test to render prognostic information to clinicians treating these patients. The control blocks for the study consisted of external positive and negative controls. The positive control had both strong and weak staining of the DCIS component and required positive reactivity of the benign terminal duct lobular units. In addition, all study cases showed positive staining of benign terminal duct lobular units. All histological sections of ER/PgR-negative breast carcinomas were reviewed by two pathologists. The histological evaluation of DCIS type, nuclear grade, presence of intraductal necrosis, and DCIS ER and PgR staining and stain intensity were evaluated. A semiquantitative scoring of percentage of positive cells with nuclear staining was used: 0 = no staining, 1+ = 1–10% staining, 2+ = 11–50% staining, and 3+ = >50% positive nuclear stain. Nuclear ER and PgR stain intensity was graded as weak, moderate, or strong.

The study was in compliance with the institutional internal review board (IRB) for which we received a waiver, as all the subjects in the study were deidentified.

## 3. Results

A total of 710 invasive breast cancer cases were collected and reviewed. One hundred and eighty-seven invasive cancers were found to be ER/PgR negative. We found a subgroup of 37 cases that had a DCIS component at least 1 mm remote from the border of invasive cancer. These cases were subjected to further study. Sixteen of the 37  ER/PgR-negative breast cancer cases (43.2%) had a DCIS component showing positive staining for ER, PgR, or both ER and PgR Tables 1 and 2. 

Upon further analysis, the subtypes of DCIS were identified as follows Table 3: 15 percent of cases were comedocarcinoma and 39.4% demonstrated mixed pattern of DCIS. Of the cases that demonstrated mixed pattern, 46% contained comedocarcinoma along with another architectural pattern of DCIS. Therefore, more than 33% of the patients were found to have comedocarcinoma or a mixed pattern containing comedocarcinoma, a subtype of DCIS with poor differentiation.

A majority of the cases (60.6%) showed a high nuclear grade Table 4. Notably, none of the cases had a low nuclear grade of DCIS. A large percentage of cases thus demonstrated a high nuclear grade and poorly differentiated subtype of DCIS suggesting that these lesions are aggressive.

## 4. Discussion

In this study of hormone-negative invasive breast cancer with concurrent noncontiguous DCIS, we demonstrate that 43.2% of the cases of DCIS were hormone receptor positive. This is in contrast to prior observations of strong concordance between hormone receptor status of breast tumors containing DCIS and contiguous invasive cancer [9, 15, 16].****

In a study by Bur et al., there was a 98% correlation between ER positivity in DCIS and the invasive component [16]. DCIS associated with an invasive carcinoma was more frequently ER positive than DCIS without invasion. All cases of ER-negative DCIS showed no ER expression in the adjacent invasive carcinoma. Barnes and Masood also reported a concordance between ER status of DCIS and the invasive component [9]. Immunohistochemical staining was positive for ER in 75% of the DCIS, 73% of DCIS with invasive cancer, and 100% of atypical hyperplasia. In 29 of 30 cases ER expression of the DCIS concurred with that found in the invasive component. 

These studies, however, only examined DCIS that occurred within the invasive cancer. In our study, we examined noncontiguous DCIS at least one millimeter apart from the invasive component. The discrepancy between the current study and previously published ones raises the question of whether ER positive and ER negative breast cancers arise from distinctly separate clones as in multifocal or multicentric disease, or evolve from one clone.

The best model for breast cancers that arise from different clones is the example of contralateral breast cancer. There have been a number of studies investigating the ER/PgR status in contralateral breast cancer [17, 18]. In the study by Arpino et al., the ER status of the primary breast cancer (PBC) was not related to the hormone receptor status of the subsequent contralateral breast cancer (CBC), suggesting that this was a stochastic stem cell event [17]. In the absence of adjuvant tamoxifen, 88% patients who had an ER-positive PBC and 75% who had an ER-negative PBC developed an ER-positive CBC (*P* = 0.11). The authors concluded that patients with an ER-negative PBC were just as likely to develop an ER-positive CBC as patients with an ER-positive PBC. Opposite conclusions were drawn by Swain et al. in another study [18]. This was a retrospective analysis of data from National Surgical and Adjuvant Breast and Bowel Project trials (NSABP) B-18, B-22, and B-25, which demonstrated that among patients who did not receive tamoxifen, 89% with an ER-positive PBC had an ER-positive CBC and 70% with an ER-negative primary breast cancer had an ER-negative contralateral breast cancer (odds ratio = 14.8, 95% *P *< 0.001). The authors concluded that the ER status of a primary breast cancer correlates with the ER status of a subsequent contralateral tumor, suggesting that another model may be operative.

It is unclear whether hormone receptor-positive and hormone receptor-negative breast cancer are derived from the same or from different stem cells. The stochastic stem cell model would support the hormone receptor-positive and hormone receptor-negative tumors derived from separate stem cells [19, 20]. A more modern theory postulates that ER-positive stem cells can evolve from ER-negative ones. Liu et al. recently showed that BRCA1 regulates human mammary stem/progenitor cell fate [21]. By using in vitro systems and a humanized NOD/SCID mouse model, they demonstrated that BRCA1 expression is required for the differentiation of ER-negative stem/progenitor cells to ER-positive luminal cells.

In our series there were no cases of low-grade DCIS. Most of the cases of ER/PgR-positive DCIS were in the intermediate grade. This is consistent with previously published studies. In the study by Bur et al. the nuclear pleomorphism was significantly correlated with absence or low percentage of ER staining in DCIS [16]. ER-positivity was more likely observed in monomorphic nuclei (*P *< 0.001). Barnes and Masood also reported that the degree of nuclear polymorphism was generally inversely related to ER positivity [9].

The finding of hormone receptor-positive DCIS in as many as 8% cases of hormone receptor-negative invasive breast cancer raises the issue of chemoprevention. Hormonal therapy is not indicated in the treatment of hormone receptor-negative invasive breast cancer. Chemoprevention, however, is widely used in the treatment of DCIS. In the 2000 Early Breast Cancer Trialists' Collaborative Group (EBCTCG) meta-analysis, compared to no adjuvant therapy, 5 years of tamoxifen was associated with a 41% relative reduction in the risk of recurrence and a 34% relative reduction in the risk of death in women with ER+/unknown breast cancers [22]. In addition, tamoxifen was associated with a 39% reduction in the annual risk of developing a contralateral breast cancer in all ER-positive women.

In a pooled analysis of NSABP trials, where tamoxifen was only given to all women above 50 years old, Swain et al. also noticed that the use of adjuvant tamoxifen appeared to reduce the risk for developing an ER-positive contralateral breast cancer [18]. However, the same study showed that 30% of patients (8 of 27 patients) with ER-negative breast tumors who were not on tamoxifen would develop an ER-positive contralateral tumor, identifying a group which may potentially benefit from hormonal therapy. It is possible that some of these ER-positive contralateral tumors may have developed from an ER-positive DCIS associated with the ER-negative invasive primary breast cancer.

In contrast to the studies in invasive breast cancer, hormonal therapy does not appear to be effective in chemoprevention in ER-negative DCIS. The NSABP B-24 trial randomized women undergoing breast-sparing surgery and radiation for DCIS, to adjuvant tamoxifen versus placebo for 5 years, irrespective of ER status [23]. There was 39% reduction of all ipsilateral and contralateral breast cancer events (16.0% in the placebo arm versus 10.0% in the tamoxifen arm; *P *= 0.0003) [24]. There was a 31% reduction in the cumulative incidence of ipsilateral breast cancers (11.1% with tamoxifen versus 7.7% with placebo, *P *= 0.02) and 47% reduction in the cumulative incidence of contra-lateral breast cancers (4.9% versus 2.3%, *P *= 0.01). Allred et al. subsequently observed that the subgroups that appears to have benefited the most were ER-positive DCIS [25]. In those subgroups tamoxifen was clearly effective (relative risk for all breast cancer events: 0.41, *P *= 0.0002). Significant clinical benefit was achieved in both the ipsilateral and the contralateral breast. In patients with ER-negative tumors, a very modest benefit was observed (relative risk for all breast cancer events: 0.80, *P *= 0.51). Data from other Tamoxifen prevention trials also showed a reduction in breast cancer events that was restricted to ER-positive tumors [26]. Based upon the NSABP B-24 results, a new clinical trial for patients with DCIS was initiated (NSABP B-35**) **[27]. Only those patients with localized ER/PgR-positive DCIS will be randomized to tamoxifen for five years or to anastrozole for five years. The primary endpoint of the study is to evaluate the effectiveness of anastrozole compared to tamoxifen in preventing subsequent breast cancer events.

In summary, the finding that ER/PgR-positive DCIS coexists in a minority of patients with ER/PgR-negative breast cancer, raises the issue of chemoprevention in this cohort of patients. Clinically, hormonal therapy is not used in the treatment of ER/PgR-negative invasive breast cancer and chemoprevention has not had demonstrated to be of benefit in ER/PgR-negative invasive breast cancer or ER/PgR-negative DCIS. However, chemoprevention is routinely used in ER/PgR-positive DCIS. The finding of ER/PgR-positive DCIS in some patients with ER/PgR-negative breast cancer may be clinically relevant and may necessitate a more careful analysis of the tissue for this setting. Confirmation of our data by other institutions, by centralized immunohistochemistry and reverse-transcription PCR (RT-PCR) [28] is warranted. 

## Figures and Tables

**Table 1:** Analysis of combined ER and PgR status of the 37 cases of intraductal breast cancer.

Hormone Receptor status	Number of patients	Percentage
ER− & PgR−	21	56.7%
ER+ & PgR+	11	29.7%
ER+ & PgR−	2	5.4%
ER− & PgR+	3	8.1%

**Table 2:** Intensity of IHC staining of DCIS.

IHC staining	ER		PR	
	Number of patients	Percent	Number of patients	Percent
0	25	68%	24	65%
1+	5	14%	6	16%
2+	2	5%	1	3%
3+	5	14%	6	16%

**Table 3:** Architecture patterns of DCIS.

Mixed patterns of DCIS	39.4%
Cribriform DCIS	21.2%
Solid DCIS	18.2%
Comedocarcinoma	15.2%
Papillary DCIS	3%
Cancerized lobule	3%

**Table 4:** Nuclear grade of DCIS.

Nuclear grade 1 (low nuclear grade)	0%
Nuclear grade 2 (intermediate nuclear grade)	39.4%
Nuclear grade 3 (high nuclear grade)	60.6%
